# Impact of *FADS* gene variation and dietary fatty acid exposure on biochemical and anthropomorphic phenotypes in a Hispanic/Latino cohort

**DOI:** 10.3389/fnut.2023.1111624

**Published:** 2023-05-05

**Authors:** Susan Sergeant, Brian A. Keith, Michael C. Seeds, Jimaree A. Legins, Caroline B. Young, Mara Z. Vitolins, Floyd H. Chilton

**Affiliations:** ^1^Department of Biochemistry, Wake Forest School of Medicine, Winston-Salem, NC, United States; ^2^Wake Forest Institute for Regenerative Medicine, Winston-Salem, NC, United States; ^3^Department of Epidemiology and Prevention, Division of Public Health Sciences, Wake Forest School of Medicine, Winston-Salem, NC, United States; ^4^School of Nutritional Sciences and Wellness, University of Arizona, Tucson, AZ, United States; ^5^Center for Precision Nutrition and Wellness, University of Arizona, Tucson, AZ, United States

**Keywords:** PUFA, highly unsaturated fatty acid, Hispanic (demographic), Latino (Hispanic), *FADS* cluster, HUFA, diet, omega-3 HUFA deficiency

## Abstract

**Introduction:**

Polyunsaturated fatty acids (PUFA) and highly unsaturated fatty acid (HUFA) synthetic products and their signaling metabolites play vital roles in immunity, inflammation, and brain development/function. Frequency differences of variants within the fatty acid desaturase (*FADS*) gene cluster affect levels of HUFAs, their biologically active products, and numerous physiological phenotypes. Fundamental questions remain regarding the impact of this genetic variation on the health of Hispanic/Latino populations.

**Methods:**

Data and biospecimens (plasma, red blood cells, buffy coat-derived DNA) from 135 participants (83.7% female) were used to assess the relationship(s) between dietary PUFA levels, a *FADS* haplotype tagging SNP, rs174537, and the capacity of Hispanic/Latino populations to generate HUFAs in plasma and RBC as well as its potential impact on anthropomorphic phenotypes.

**Results:**

The dietary habits of the cohort showed that participant diets contained a high ratio (9.3 ± 0.2, mean ± SEM) of linoleic acid (*n*−6) to alpha-linolenic acid (*n*−3) and also contained extremely low levels of *n*−3 HUFAs (eicosapentaenoic acid, EPA and docosahexaenoic acid, DHA), both features of the Modern Western Diet. Compared to African and European American cohorts, the frequency of the TT rs174537 genotype was highly enriched (53% of subjects) in this Hispanic/Latino cohort and was strongly associated with lower circulating HUFA levels. For example, plasma levels of arachidonic acid (ARA: 20:4, *n*−6) and EPA (20:5, *n*−3) were 37% and 23%, respectively, lower in the TT versus the GG genotype. HUFA biosynthetic efficiency, as determined by metabolic product to precursor ratios, was highly dependent (*p* < 0.0001) on the rs174537 genotype (GG > GT > TT) for both circulating *n*−6 and *n*−3 HUFAs. In contrast, the RBC Omega-3 Index (EPA + DHA) was extremely low (2.89 ± 1.65, mean ± sd) in this population and independent of rs174537 genotype. Importantly, the rs174537 genotype was also related to female height with TT genotype participants being 4.5 cm shorter (*p* = 0.0001) than the GG + GT participants.

**Discussion:**

Taken together, this study illustrates that dietary PUFA + HUFA × *FADS* gene- interactions place a large proportion (>50%) of Hispanic/Latino populations at high risk of a deficiency in both circulating and cellular levels of *n*−3 HUFAs.

## Introduction

**Figure 1:**
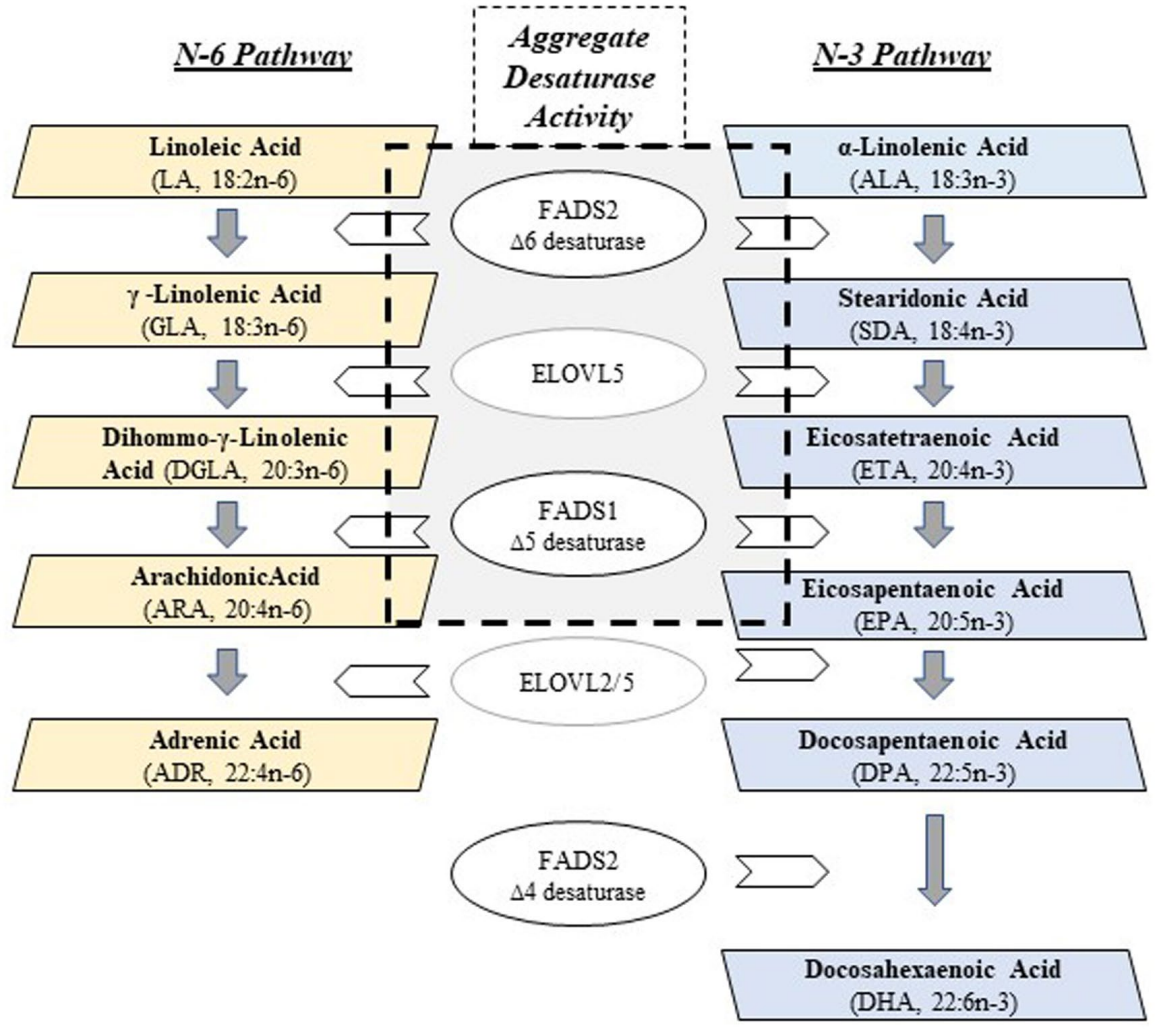
PUFA + HUFA Synthetic Pathway. *N*−3 and *n*−6 PUFA+HUFA synthesis proceeds in parallel, using shared and alternating fatty acid desaturase (FADS) and elongation (ELOVL) enzymatic steps. The essential medium chain fatty acids (18 carbons, PUFAs), linoleic (LA, *n*−6) and alpha-linolenic (ALA, *n*−3), must be obtained from the mammalian diet in order to be converted to HUFAs. The central gray box denotes the enzymatic steps (FADS2, ELOVL5, FADS1) that collectively, will be referred to as Aggregate Desaturase Activity, an estimate of PUFA+HUFA metabolic efficiency: ARA/LA (*n*−6) and EPA/ALA (*n*−3), the product/essential PUFA precursor ratios.

Most (>90%) polyunsaturated fatty acids (PUFA) are obtained from plant-derived dietary sources as 18 carbon (18C) PUFAs, such as linoleic (LA, 18:2*n*−6) and alpha-linolenic acid (ALA, 18:3*n*−3). They are enzymatically converted by alternating desaturation and elongation steps (Figure 1) in tissues, primarily in liver, to *n*−3 and *n*−6 highly unsaturated fatty acids (HUFA; containing ≥3 carbon–carbon double bonds). The two desaturase enzymes, Δ5 and Δ6 desaturase, are encoded by *FADS1* and *FADS2*, respectively, and the two elongation enzymes, elongase 2 and elongase 5, by *ELOVL2* and *ELOVL5* (1–3). *n*−6 and *n*−3 HUFAs produced by this pathway include dihomo-gamma linolenic acid (DGLA, 20:3*n*−6), arachidonic acid (ARA, 20:4*n*−6), eicosapentaenoic acid (EPA, 20:5*n*−3) and docosahexaenoic acid (DHA, 22:6*n*−3). There are exceptions, but in general, ARA is converted to pro-thrombotic/pro-inflammatory metabolites and DGLA, EPA and DHA to anti-inflammatory/pro-resolving/anti-thrombotic metabolites. The HUFAs themselves and their many signaling metabolites, including oxylipins and endocannabinoids, play vital roles in regulating innate immunity, inflammation, brain development/function, thrombosis and energy metabolism.

The multiple levels of regulation of the PUFA pathway has been a topic of much investigation. At the substrate level, *n*−6 and *n*−3 PUFAs (dietary intermediates) compete throughout the synthetic pathway, and the Δ5 desaturase has recently been identified as a key rate-limiting step through the PUFA pathway (Figure 1) (4). This competition is important, given the dramatic increase in the ingestion of LA (*n*−6)-containing cooking oils and processed foods has resulted in 6 to 9% of daily energy coming from LA alone. Thus, the LA to ALA ratio typically entering the pathway is >10:1 (5, 6).

Until recently, the prevailing belief was that the capacity of humans to synthesize HUFAs from dietary precursors (LA and ALA) was uniform in all human populations. More recent studies indicate that there are dramatic ancestry-based frequency differences in genetic variation within the *FADS* gene cluster (human Chr11q), giving rise to divergences in the capacity of individuals to synthesize *n*−6 and *n*−3 HUFAs (7–10). For example, an ancestral *FADS* haplotype, associated with a limited capacity to synthesize HUFAs is nearly fixed in Native American and Greenland Inuit populations (11). However, this same haplotype is virtually absent and replaced by a derived *FADS* haplotype in African populations, and approximately 80% of African Americans and 44% of European Americans have two *FADS* alleles with the derived haplotype (11, 12). It has been shown that positive selection for the *FADS* haplotype toward the derived or ancestral haplotypes was driven by the need for ancient humans to adapt to environments that offered dramatically different levels of dietary PUFAs (11, 12).

These marked differences in *FADS* genetic variations in early human populations have persisted in modern populations and now challenge the “one size fits all” dietary PUFA recommendations in geographic areas such as the US where there is great population diversity. It is important to point out that the *FADS* cluster not only regulates levels of HUFAs and their metabolites, but this region has recently been suggested to be the largest multi-morbidity-associated gene cluster in the genome associated with over 40 clinical/disease phenotypes that are related to fat and lipid metabolism, inflammatory diseases/disorders and numerous cancers (13).

Combining a metabolomic and genome-wide associations study analyses, we demonstrated that the FADS1 step was associated with 23 HUFA-containing lipids and signaling molecules, including unesterified fatty acid, phospholipids, lyso-phospholipids and the endocannabinoid, 2-ARA-glycerol (4). Perhaps most surprising was that of all HUFA-containing complex lipids and signaling molecules examined, there was no other significant genetic association outside of the *FADS* cluster. Together, these studies reveal the importance of both dietary PUFA and HUFA exposure and genetic variation across the *FADS* cluster to the balance of *n*−6 and *n*−3 HUFAs and their metabolites that have the potential to impact human clinical/disease phenotypes.

In 2016, the Hispanic/Latino population in the US reached ~61 million, making Hispanics the largest racial/ethnic minority. Hispanic/Latinos have higher rates of obesity, poorly controlled high blood pressure, elevated circulating triglycerides (TGs), along with a higher prevalence of diabetes and nonalcoholic fatty liver disease (NAFLD) (14, 15). Importantly, self-identified Mexican Americans (MxAm) have the highest levels of Amerind (AI) ancestry (genetically related to the indigenous peoples of the Americas) among all US Hispanic/Latino populations (16), and AI-ancestry Hispanic/Latino populations are 2-fold more likely than other Hispanics to have NAFLD. We recently examined the US-based Multi-Ethnic Study of Atherosclerosis (MESA) cohort, which includes participants from Central America, South America, Mexico, Dominican Republic, Cuba and Puerto Rico, and demonstrated that individuals with high Amerind ancestry resulted in low (perhaps inadequate) levels of *n*−3 HUFAs, including EPA and DHA in circulating phospholipids (17).

For this study, the Latinos Combating Diabetes Study provided an opportunity to conduct a secondary evaluation of the interaction between dietary PUFA and *FADS* variant interactions in a largely Mexican American cohort. This parent study was a weight-loss intervention study with community and lifestyle-based interventions built on existing infrastructure in a local the Hispanic/Latino community. The goal of the parent study was to explore engagement techniques to attain a clinically meaningful impact on HbA1c, insulin metabolism, and markers of the metabolic syndrome. Using only the baseline data from the parent study, we sought to understand relationships between dietary PUFA exposure and *FADS* genetic variants on plasma (circulating) and RBC levels of PUFA and HUFA, as well as previously identified anthropomorphic phenotypes.

## Materials and methods

### Study population

The Latinos Combating Diabetes Study (ClinicalTrials.gov Identifier: NCT01831921) was designed to recruit adults (≥18 years old) who self-identified as Hispanic or Latino (and an absence of African American, Native American, Asian or White ancestry) and had evidence of pre-diabetes (Hemoglobin A1C of 5.6%–6.5%). Briefly, participants of this parent study were randomized to one of two intervention arms to evaluate intervention impact on Hemoglobin A1C and weight loss. While both interventions were conducted in a community setting, one arm was an intensive lifestyle intervention, and the control arm was usual care. Pre-existing major disease (diabetes, any type of cardiovascular disease, cancer) or pregnancy was exclusionary. This study, including informed consent documents in both English and Spanish, was reviewed and approved by the Institutional Review Board of Wake Forest University School of Medicine. All participants were required to provide written informed consent prior to taking part in any study-related data collection. For regulatory purposes, consent for the collection of biological samples was documented in a separate section of the consent form denoting that this as a sub-study. Only participants who signed this additional section in the consent document for the use of their biological samples were considered for this analysis.

Only baseline biospecimens and data were utilized in the current study. Fasting blood (8 h overnight fast) was collected in the Clinical Research Unit of the Wake Forest Baptist Medical Center. Biospecimens were stored frozen (−70°C) until analyses. Table 1 shows the demographic characteristics and blood chemistries of the 135 participants evaluated for the current study (for whom buffy coat samples were available for DNA isolation and who unequivocally self-identified as solely Hispanic/Latino).

**Table 1:** Demographics and clinical values of the Hispanic/Latino cohort at baseline.

Cohort size (*n*)	135	% female	83.7
			
	**Mean (SD)**	**95% CI**	**Unit**
Age	41.3 (8.9)	39.8–42.8	Years
Height	157.4 (7.9)	156.1–158.8	cm
Weight	81.3 (13.6)	78.9–83.6	kg
Waist	103.0 (11.7)	101.0–105.0	cm
BMI	32.9 (4.5)	32.1–33.6	
Glucose	97.3 (9.0)	95.8–98.9	mg/dL
Insulin	18.9 (9.2)	17.3–20.5	μU/mL
HOMA-IR	4.6 (2.4)	1.2–5.0	na
HbA1C	6.0 (0.3)	5.9–6.0	%
TG	145.5 (67.2)	134.0–156.9	mg/dL
Total Cholesterol	182.8 (33.2)	177.1–188.4	mg/dL
VLDL	29.1 (13.4)	26.8–31.4	mg/dL
LDL	108.7 (27.2)	104.1–113.3	mg/dL
HDL	45.0 (11.1)	43.1–46.9	mg/dL

The cohort composition is shown along with baseline anthropomorphic indices and fasting blood chemistries. HOMA-IR was calculated using the formula: (mg glucose/mL × μU insulin/mL)/405. Data are presented as the mean with standard deviation with 95% confidence intervals (CI).

**Figure 2:**
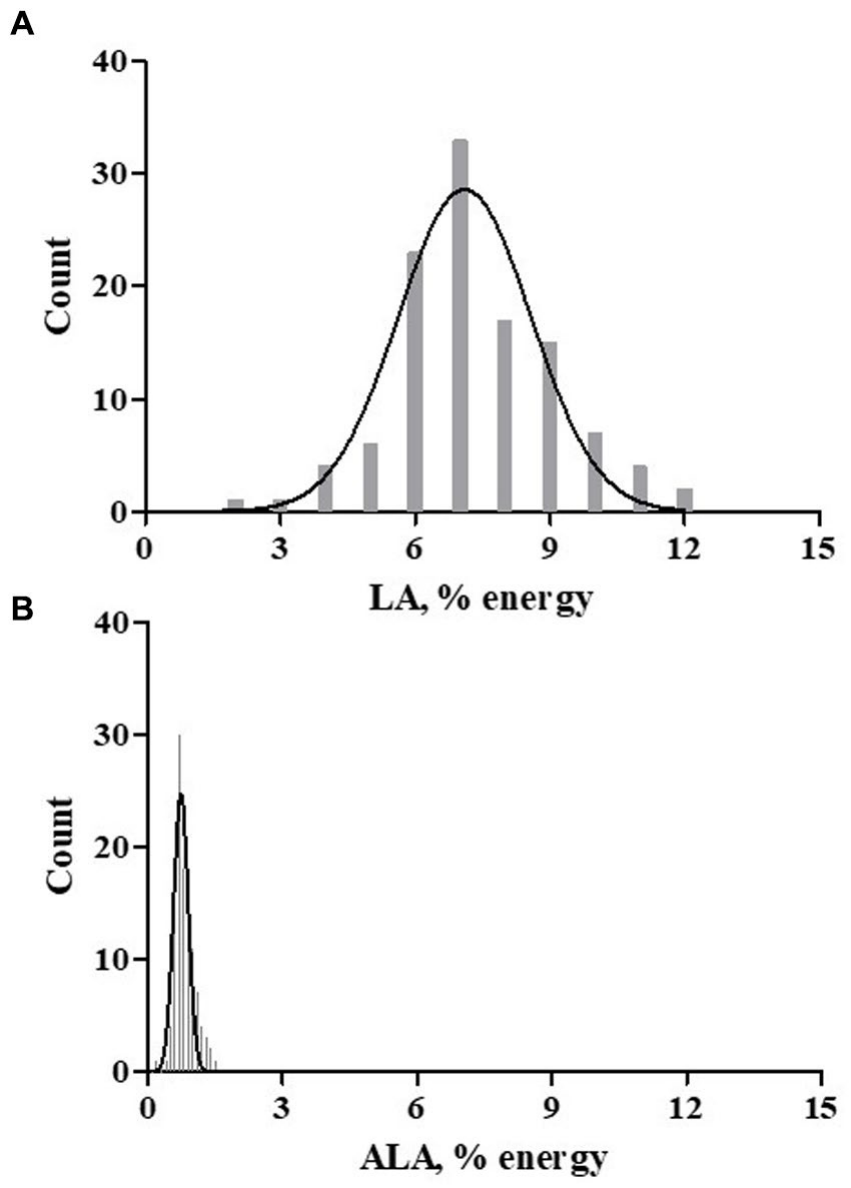
Dietary LA and ALA Exposure. A Spanish language version of the Dietary History Questionnaire-1 (DHQ-1) was used to capture nutrients exposure based on the typical dietary patterns. DHSQ-1 data from 113 participants was analyzed. The dietary essential fatty acids exposure histograms for LA **(A)** and ALA **(B)** are shown as percent of daily energy (kcal).

A European ancestry cohort of healthy adults, residing in the same area of North Carolina as the Hispanic/Latino cohort, was used only as a comparator for the frequency distribution of the *FADS* gene cluster variant rs174537 (Figure 2). This cohort (*n* = 100; 71% female) was a subset from a larger non-interventional study that was reviewed and approved by the Wake Forest Baptist Medical Center Institutional Review Board (IRB00016822). This subset cohort was ill-suited for further comparison with the Hispanic/Latino cohort as the European ancestry cohort was younger (37.1 ± 11.6; mean, SD), had a considerably lower mean BMI (23.9 ± 2.8) and did not have pre-diabetes (an exclusion criterion for that study).

### Blood chemistry measurements

Fasting blood (8 h overnight fast) was collected from the Hispanic/Latino cohort participants and used for the measurements of serum lipids [Total Cholesterol, HDL, LDL, TG, VLDL(calculated)] and glucose-regulatory variables (glucose, insulin, HbA1C, HOMA-IR). These measurements were performed by LabCorp, a third party clinical testing laboratory (Burlington NC, United States). Additional tubes of blood were obtained for DNA isolation (in acid citrate dextrose) and for fatty acid analyses of plasma and red blood cells (in EDTA).

### DNA isolation and SNP genotyping

Leukocyte-derived DNA was isolated from whole, frozen blood by routine molecular biology techniques (Gentra Puregene, Qiagen, Germantown MD, United States) and qualified by UV absorbance. This DNA was used to determine the rs174537 genotype employing a PCR-based TaqMan methodology. Following the manufacturer’s instructions, DNA (5 μg) was incubated in the presence of a TaqMan Universal Master Mix II (Applied Biosystems; Waltham MA, USA) and validated rs174537 primers (Applied Biosystems) in an Applied Bioscience 7,500 PCR instrument. Negative and positive controls (derived cell line-derived DNA) were employed.

### Analysis of plasma and red blood cells fatty acids

Fasting baseline plasma was utilized for the analysis of circulating fatty acids by a method previously described (18), which was modified from that originally reported by Metcalf (19). Briefly, fatty acids in duplicate plasma samples (100 μL) were hydrolyzed from complex lipids under alkaline conditions in the presence of an internal standard (triheptadecanoin; 100 μg; NuChek Prep; Elysian MN, United States). Fatty acids were then converted to methyl esters in the presence of boron trifluoride (Fisher Scientific; Waltham MA, United States). The fatty acid methyl esters (FAMEs) were identified on the basis of retention times of commercially available authentic fatty acid methyl ester standards and quantified relative to the internal standard. Approximately 25 fatty acids accounted for >99% of the total fatty acids in the sample.

Total sample fatty acids were analyzed by gas chromatography with flame ionization detection using either a Hewlett Packard 5,890 or 7890B (Agilent Technologies; Santa Clara CA, United States) instruments with identical results based on their respective response factor. Each response factor was calculated using external standard (fatty acid methyl esters of 16:0, 18:0, 18:1, 18:2, 10:0, 20:5, 22:0, 22:5, 23:0, 24:0) sets for quality assurance purposes (20). A mixture of known FAMEs was run with each sample set to monitor instrument performance. Both instruments were equipped with an Agilent J&W DB-23 column (30 m, 0.25 mm ID, 0·25 μm film; Agilent) fitted with an inert pre-column (1 m, 0.53 mm ID) for cool on-column injection.

After blood collection, red blood cells (RBC) were washed with isotonic saline, suspended to approximately 50% hematocrit, aliquotted and then stored at −70°C. RBC fatty acid content was analyzed in thawed cell lysates. A total lipid extract was prepared from RBC lysates (400 μl in duplicate) using an acidified Bligh-Dyer method (21). The resultant chloroform extract was dried under a stream of nitrogen in the presence of the internal standard (50 μg triheptadecanoin). This extract was then subjected to alkaline hydrolysis and derivatization for FAME preparation and analyzed as described for plasma above. To account for differences in the RBC content of the lysates, the fatty acid data was normalized to the hemoglobin content (below) of the lysate.

The fatty acid data are presented as percent of total fatty acids derived from concentration data for plasma (mg fatty acid/dl plasma). RBC fatty acid content was normalized to hemoglobin mass (mg fatty acid/g hemoglobin).

### Determination of hemoglobin concentration

The hemoglobin content of RBC lysates was quantitatively determined using a colorimetric (Drabkin) method (22, 23) following the manufacturer’s (Stanbio Laboratory; Boene TX, United States) instructions. Duplicate RBC lysate samples were diluted in the Drabkin reagent and absorbance at 540 nm was recorded. The lysate hemoglobin content (mg/mL) was calculated from a cyanmethemoglobin standard curve.

### Dietary exposure assessment

The Spanish version of the Diet History Questionnaire-1 [DHQ1; (24)] was administered to study participants in a paper format at baseline. A subset of 119 participants completed the 144-item questionnaire. Sixteen participants declined to complete the dietary survey. The response data were captured by manual data entry and analyzed with the NCI Diet*Calc software program [v1.4.3; (24)] in order to extract dietary fat calories and dietary fatty acid exposures, specifically total PUFAs, essential fatty acids (LA, ALA) and HUFAs (ARA, EPA, DHA). Although this is an older food frequency questionnaire instrument, it has been validated under multiple conditions (25–28) and it is the first that could be offered in the Spanish language.

### Data analysis

Statistical analyses were performed using R version 4.1.3 (29), Stata11 and GraphPad Prism5 (San Diego CA, United States). Data were checked for outliers. Descriptive statistics were calculated as mean, standard deviation (SD) and 95% confidence intervals (95% CI). Comparison of rs174537 genotype were tested linear regression using an additive model for genotype with adjustments for fatty acid intake and for multiple testing (Bonferroni). In certain cases, non-parametric Kruskal-Wallis ANOVA with Mann–Whitney U *post hoc* analysis was employed. When a recessive model (GG + GT vs. TT) was employed, the Students t test (two-tailed) was employed. Correlations were evaluated using Spearman analysis.

## Results

An assessment of dietary fatty acid exposure in this cohort was accomplished using a validated food frequency questionnaire administered in Spanish. The hardcopy Diet History Questionnaire-1 (DHQ1; 144 items) was attempted by 119 participants at the baseline visit. Of these 119 dietary exposure questionnaires, the data from 113 participants was further evaluated for nutrient exposures. The questionnaires from 6 participants were rejected by the analysis software [Diet*Calc, (24)] based on its criteria for data completeness. The survey was used to capture nutrient estimates (typical food choices, frequency and portion size) and dietary supplement use. With regard to PUFA-related supplements, 16 participants (14%) reported the use of fish oil and two participants (1.7%) reported the use of GLA-enriched products (evening primrose oil or spirulina). The assessment of typical dietary habits showed a striking difference in dietary exposure to the essential fatty acids, LA (*n*−6) and ALA (*n*−3), expressed as % of daily energy (kcal), as is evident in Figure 2. The dietary intake pattern of the participants of the current study was not unique, as it was comparable to that of a recent, and much larger assessment of Hispanics living in the US, as shown in Table 2 (30). LA comprised 7.29% of daily energy and ~ 90% of all PUFA+HUFAs consumed in the diet. In both assessments, the ratio of LA/ALA dietary exposure had a value greater than 9. This suggests that Hispanic/Latino populations residing in the US are typically consuming the Modern Western Diet. An evaluation of the genetic variants of the *FADS* gene cluster in this cohort is expected to be informative since this gene cluster governs the PUFA pathway efficiency and as such will be instructive regarding how well-suited Hispanic/Latino populations are to the Modern Western Diet.

**Table 2:** Dietary fat exposure evaluated in Hispanic/Latino cohorts.

		Current study		NHANES^a^	
		Mean	SEM	Mean	SEM
Energy (kcal/day)	95% CI	2,139.84	114.00	2,008.00	22.00
		1,922, 2,374			
Fat (%E)	95% CI	34.98	0.65	33.70	0.30
		33.70, 36.27			
Fat (g/day)	95% CI	86.40	5.50	76.90	1.70
		75.5, 97.26			
PUFA (g/day)	95% CI	20.04	1.27	17.60	0.30
		17.51, 22.58			
LA (g/day)	95% CI	17.90	1.15	15.60	0.20
		15.64, 20.20			
Linolenic Acid^b^ (g/day)	95% CI	1.93	0.10	1.65	0.03
		1.7, 2.16			
ARA (g/day)	95% CI	0.15	0.11	na^c^	na
		0.13, 0.17			
EPA (g/day)	95% CI	0.03	0.04	0.02	0.001
		0.02, 0.04			
DHA (g/day)	95% CI	0.07	0.01	0.05	0.003
		0.06, 0.09			
LA (%E)	95% CI	7.29	0.17	6.80	0.10
		6.96, 7.62			
ALA (%E)	95% CI	0.81	0.02	0.72	0.01
		0.77, 0.85			
LA/ALA	95% CI	9.30	0.20	9.45^d^	
		8.91, 9.67			

Dietary fat exposure for the current study (*n* = 113) was evaluated by the Dietary History Questionnaire-1 (DHQ-1) and in US-dwelling Hispanics by the NHANES 2015–2018 survey [*n* = 4,186; (30)]. Data are the mean ± SEM with 95% confidence interval (current study only). Units for the nutrients are indicated parenthetically.

a Data from the 2015–2018 What We Eat in America database for Hispanics living in the US.

b Assumed to be ALA intake.

c Data not available.

d Estimate based on this table gram values for LA and ALA.

**Figure 3:**
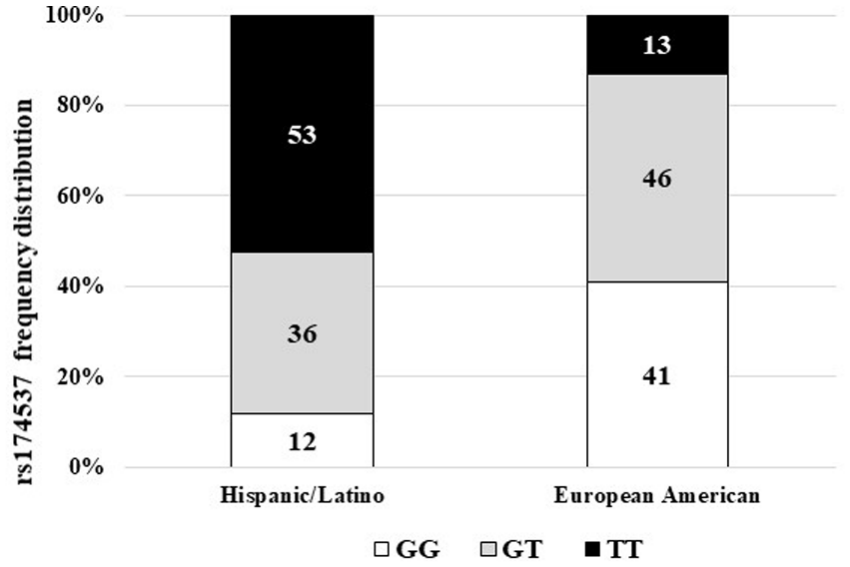
Frequency distribution of FADS Variant rs174537 in Hispanic/Latino and European American Cohorts. Leukocyte-derived DNA was genotyped at rs174537 using a TaqMan platform, as described in Methods. The frequency of the genotype distribution for the Hispanic/Latino cohort and a European American cohort living the same geographical area are shown.

Over the past decade, we have explored population differences in the genetic variants of the *FADS* gene cluster, the impact of these variants on the metabolism of HUFAs (8, 9), and the role(s) that these variants may play in the differences in disease burden for different populations (10, 17, 31). An assessment of the frequency distribution of the rs174537 SNP in this cohort (*n* = 135), self-identifying solely as Hispanic/Latino, was unlike any we have previously analyzed. Figure 3 shows that the frequency distribution for rs174537 in this Hispanic/Latino group had a profile nearly opposite of that typically observed for a European American ancestry cohort residing in the same geographical region. Most notably, the TT genotype predominates (53%) in the Hispanic/Latino cohort. This same genotype has a very low frequency (~10%) in populations of European ancestry (Figure 3) and is exceedingly rare in African Americans [<1%; (8)].

**Figure 4:**
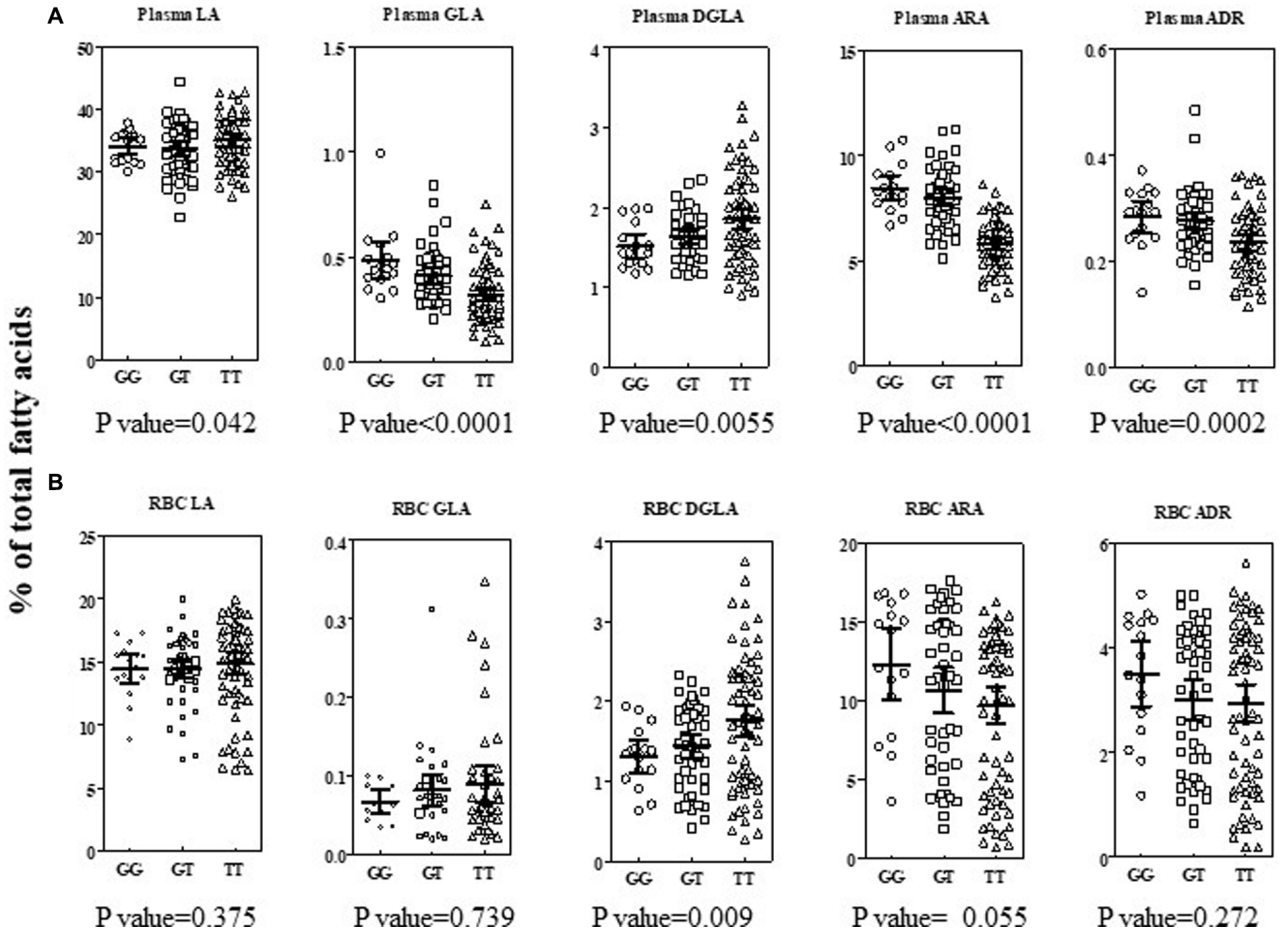
Impact of rs174537 on *n*−6 PUFA + HUFAs in Plasma and RBC in a Hispanic/Latino cohort. Total plasma (**A**, top row) and RBC (**B**, bottom row) fatty acid were analyzed as described in Methods and expressed as percent of total fatty acid in the sample. Individual data are shown, stratified by rs174537 genotype (○, GG; □, GT; Δ, TT) for each *n*−6 PUFA along the PUFA+HUFA pathway. The mean and 95% CI is shown for each genotype. The data were analyzed by linear regression using an additive model for genotype, adjusting for fatty acid intake and multiple testing (Bonferroni correction, *p* = 0.0029). The resultant *p*-value for impact of genotype on each fatty acid stated under each plot.

Previously, we showed that the rs174537 TT genotype is associated, phenotypically, with a low efficiency of PUFA biosynthetic pathway in European American and African American cohorts (8, 9). Therefore, we sought to evaluate the impact of the *FADS* genotypic frequency on circulating fatty acid profiles in this Hispanic/Latino cohort. These relationships have typically been assessed in the fasting plasma fatty acid pool. However, the availability of matched red blood cells samples afforded a novel opportunity to apply this evaluation in a second easily accessible tissue and in parallel to plasma. Figure 4 shows the *n*−6 PUFA levels, stratified by rs174537 genotype and adjusted for fatty acid intake, for plasma (Figure 4A) and for RBC (Figure 4B). It is notable that the LA content in both tissues was elevated, which reflects the consumption of a typical LA-enriched Modern Western Diet. Only the circulating (plasma) level of ARA was modestly impacted by dietary intake of ARA after adjustment for multiple testing (*p* = 0.001; Bonferroni correction, *p* = 0.0029). Nevertheless, each of the LA-derived *n*−6 HUFAs (GLA → DGLA → ARA) products in the plasma of this Hispanic/Latino cohort demonstrated a clear genotypic-dependence. This pattern is analogous to that seen in European and African ancestry cohorts with highest plasma ARA levels in the GG genotype and the lowest in the TT group (Figure 4A).

Similar to the observation for most plasma fatty acid levels, no significant impact of dietary fatty acid intake (as assessed by dietary survey) was seen for RBC fatty acid levels. However, in contrast to plasma, a genotypic effect on RBC *n*−6 PUFA+HUFA content was not generally evident (Figure 4B). In RBC, there was a trend for a genotypic impact for ARA content, but the wide variation in the content of this HUFA likely reflects a longer-term variation in dietary exposure to ARA and to a lesser extent the impact of hepatic PUFA metabolism. When a recessive model (GG + GT vs. TT; data not shown) was employed, then a genotypic impact for RBC DGLA (two-tailed t test; *p* = 0.01) and RBC ARA (two-tailed t test; *p* = 0.04) were observed. It is noteworthy that the PUFA+HUFA content of RBC was on average 25% lower than that of plasma. The lower RBC PUFA+HUFA content was compensated for a larger saturated fatty component compared to plasma.

The levels of *n*−3 PUFA+HUFAs were, on average, 14- and 8-fold lower than the *n*−6 PUFA+HUFA content of plasma and RBC, respectively (Supplementary Figure 1A). No impact of dietary fatty acid intake was observed to affect *n*−3 PUFA or HUFAs after adjustment for multiple testing. However, the levels of two plasma *n*−3 HUFAs were impacted by rs174537 genotype (EPA, *p* = 0.001; DPA, *p* = 0.0001). EPA and ARA are both products of FADS1 in the PUFA synthetic pathway (Figure 1). DPA is the elongation product of EPA and parallel to adrenic acid (ADR, 22:5 *n*−6) on the *n*−6 side of the PUFA pathway. Collectively, the plasma PUFA+HUFA patterns in this rs174537 TT-enriched Hispanic cohort align with those previously observed for European and African ancestry cohorts. In contrast, neither RBC *n*−6 HUFAs nor *n*−3 HUFA profiles were significantly impacted by rs174537genotype (Supplementary Figure 1B) or dietary intake.

**Figure 5:**
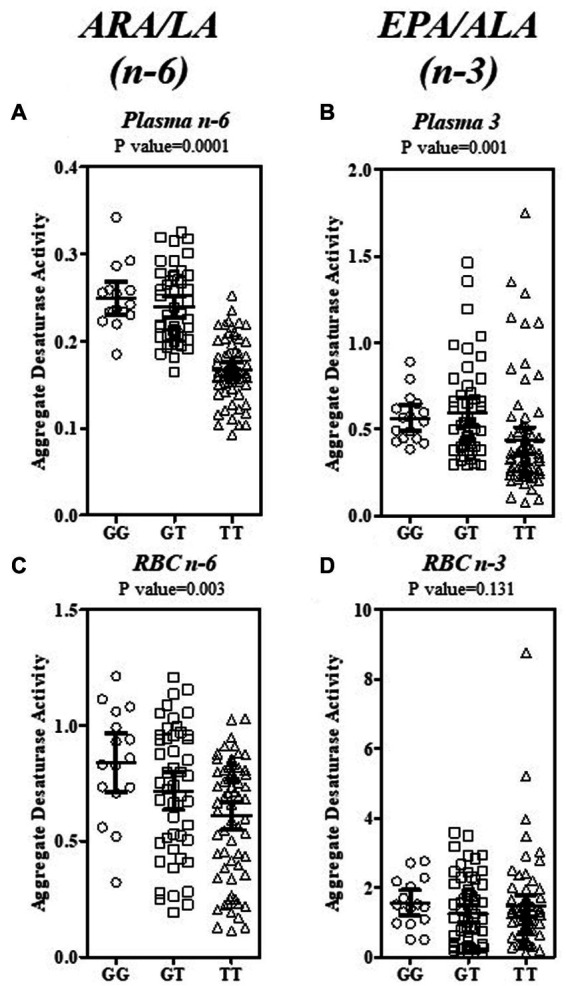
Estimation of the Aggregate Desaturase Activity in the PUFA + HUFA Pathway for Plasma and RBC in a Hispanic/Latino Cohort. The Aggregate Desaturase Activity (FADS2 → ELOVL2 → FADS1) of both the *n*−6 and *n*−3 sides of the PUFA + HUFA pathway was estimated by the product—precursor ratio surrogates of ARA/LA (*n*−6) and EPA/ALA (*n*−3). The ratios for plasma (**A**, *n*−6; **B**, *n*−3; top row) and RBC (**C**, *n*−6; **D**, *n*−3; bottom row) are shown as individual ratio data, stratified by rs174537 genotype(○, GG; □, GT; Δ, TT). The mean and 95% CI is shown for each genotype. The data were analyzed by ANOVA with the resultant *p*-value for each fatty acid ratio stated above each plot.

Fatty acid product-precursor ratios are typically utilized as a surrogate for PUFA pathway enzymatic steps. Figure 5 shows the ratios for the Aggregate Desaturase Activity (FADS2 → ELOVL2 → FADS1; Figure 1 central gray box), which represents the enzymatic step common to the *n*−6 (ARA/LA) and *n*−3 (EPA/ALA) sides of the PUFA pathway utilizing the same enzymes to service both substrate types. The plasma ratios for both the *n*−6 and *n*−3 Aggregate Desaturase Activities, were robustly differentiated by rs174537 genotype (*p* = 0.0001, *p* = 0.0001, respectively; Figures 5A,B; Supplementary Table 1). This is consistent with the gradient of high (GG) to low (TT) metabolic efficiency of the PUFA pathway governed by this genotypic variant (8, 9).

In contrast, the hardiness of the genotypic governance of the RBC aggregate ratios were more modest for *n*−6 (ARA/LA, *p* = 0.003; Figure 5C) than that for plasma and was absent for the RBC *n*−3 EPA/ALA ratio (*p* = 0.131; Figure 5D). In order to determine the enzymatic step(s) that contributed to this genotypic governance, a ratio surrogate was calculated for each of the FADS and ELVOL steps within the Aggregate Desaturase and beyond (Supplementary Table 1). The abundance of the *n*−6 PUFA+HUFA lent itself to assessment of each PUFA pathway step. Plasma estimates of FADS2 (GLA/LA), ELVOL5 (DGLA/GLA) and FADS1 (ARA/DGLA) activities are all strongly associated with rs174537 genotype. It was not possible to quantitate plasma SDA or ETA (eicosatetraenoic acid, 20:4*n*−3) levels across this cohort due to their low abundance. Thus, it is not possible to confirm, unequivocally, that these three enzymatic steps are similarly genetically governed on the *n*−3 PUFA side of the pathway. Interestingly, the impact of rs174537 on plasma Δ6 FADS2 activity (GLA/LA, *n*−6; *p* < 0.0001) was much more robust than that for its plasma Δ4 activity (DHA/DPA; *n*−3, *p* = 0.0698).

RBC have recently been observed to express very low levels of FADS1/2 protein and activity, which may function as a compensatory mechanism to maintain RBC integrity (32). The product/precursor activity surrogate, ARA/DGLA, for FADS1 step explains the majority (p < 0.0001) of the genotypic impact on the *n*−6 PUFA Aggregate Desaturase Activities in this tissue (Figure 5C; Supplementary Table 1). It remains to be determined the extent to which FADS1 activity (hepatic vs. RBC) predominates in defining the RBC HUFA profile. ELOVL2 (DGLA/GLA) activity provides a small (*p* = 0.0107), but significant contribution to the RBC *n*−6 PUFA pathway. A genotypic impact on *n*−3 PUFA activity (EPA/ALA; *p* = 0.0621) was absent when estimated from RBC PUFA levels.

Beyond the core Aggregate Desaturase Activity surrogate, only the subsequent ELOVL2/5 step (ADR/ARA; *p* = 0.001; Supplementary Table 1) on the *n*−6 PUFA side showed a contribution to genetic governance by the *FADS* variant. The plasma DHA/EPA (not shown), which utilizes both elongation and desaturase steps (Figure 1), was modestly impacted (ANOVA, 10.52 (2), *p* = 0.0052) by rs174537. This examination of the PUFA pathway surrogates in blood components suggests that the plasma PUFA profile is under more immediate influence of hepatic PUFA pathway efficiency regulation than that for the RBC PUFA profiles, which are thought to be a reflection of longer-term diet exposure.

Given the relatively low abundance of *n*−3 PUFA+HUFA blood levels in this cohort, an evaluation of the Omega-3 Index (sum of RBC EPA + DHA) was of particular interest. The Omega-3 Index has been proposed to be a useful marker of *n*−3 HUFA status (33, 34) and a potential disease risk factor (35, 36). Although the Omega-3 Index did not differ by rs174537 genotype (Supplementary Figure 2), the mean cohort value was 2.89 (±1.65, SD), which is in the range (<4%) that is suggested to be the least protective against cardiovascular disease (36).

**Figure 6:**
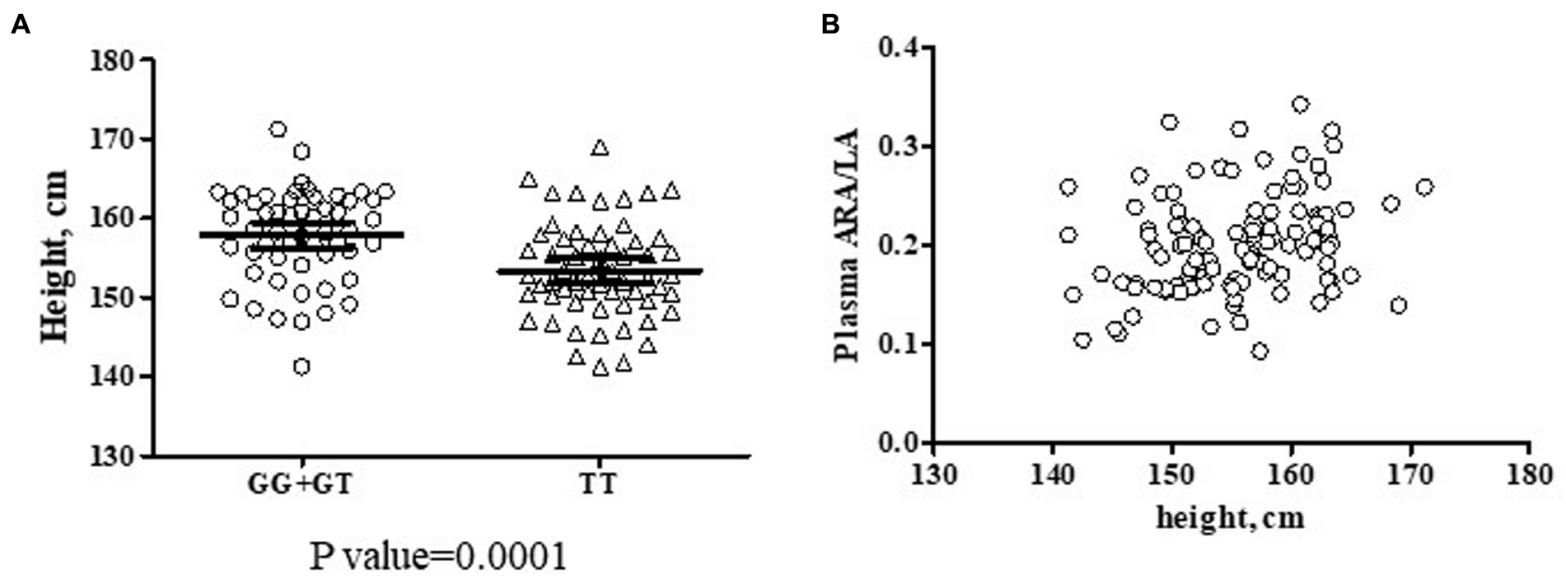
Potential Interactions Among rs174537, Female Height and Aggregate Desaturase Activity in a Hispanic/Latino Cohort. **(A)** Two female subsets of the cohort were created based on the presence of the rs174537 G allele (GG + GT, ○, *n* = 54; and TT, Δ *n* = 59) in order to evaluate a genotypic impact on participant height. The data were analyzed by two-tailed t test with the resultant *p*-value for the subset comparison stated under the plot. **(B)** An association of female height (*n* = 113) with *n*−6 Aggregate Desaturase Activity (ARA/LA) was evaluated, Spearman *r* = 0.2652, *p* = 0.0045.

Beyond the biochemical phenotypes, clinical phenotypes have also been evaluated in the context of *FADS* gene variants. Only height was significantly impacted by rs174537 genotype. Across the entire cohort (*n* = 135), participants carrying the GG genotype were, on average, 6.5 cm taller (*p* = 0.0036, Supplementary Table 2) than those having the TT genotype at rs174537. However, a sex difference was observed since males (*n* = 22) were significantly taller than females (*n* = 113; *p* < 0.0001, two-tailed t test) so height was examined more closely. In order to eliminate the influence of sex, the impact of *FADS* genotype on height was re-evaluated in the female subset. Figure 6A shows that the female TT participants (*n* = 59) were clearly of shorter stature (on average a 4.5 cm difference; *p* = 0.0001, two-tailed *t*-test) than those carrying the G allele (GG + GT; *n* = 54). Although body weight would be expected to be impacted more directly by lifestyle choices, female body weight was also significantly lower in TT subjects compared to those carrying the G allele (TT, 76.9 ± 1.6 kg [73.8–79.9, 95%CI]; GG + GT, 82.2 ± 1.7 kg [78.8–85.6, 95%CI]; *p* = 0.0216, two-tailed t test). Finally, a positive relationship between height and the *n*−6 Aggregate Desaturase Activity surrogate (ARA/LA) was observed (Figure 6B) for the female subset of the cohort (Spearman *r*, 0.2652; 95%CI 0.0791 to 0.4335; *p* = 0.0045). Interestingly, when taking into account each individual PUFA pathway step, the association with height was more robust for FADS1 activity (ARA/DGLA; Spearman *r*, 0.2427; 95%CI: 0.0552 to 0.4137; *p* = 0.0096) than for FADS2 activity (GLA/LA; Spearman *r*, 0.17152; 95%CI: −0.0192 to 0.502; *p* = 0.069).

## Discussion

The frequency distribution of rs174537 in the pre-diabetic Hispanic cohort was found to be strikingly different from that of European or African ancestry cohorts, but quite similar to that observed for a Mexican-dwelling cohort (37) and other populations of South and Central American ancestries (17, 38, 39). The rs174537 variant is in high linkage disequilibrium (LD) with a number of other *FADS* SNPs (38) that have been evaluated in Hispanic populations and would be expected to behave similarly. These ancestry-related differences in *FADS* variants, which govern PUFA+HUFA biochemical metabolic phenotypes, give rise to important considerations concerning gene-diet interactions that may contribute to health disparities.

Our analyses of the genotypic-dependence of *n*−3 and *n*−6 PUFA+HUFAs showed that circulating levels of desaturase step products and precursors were significantly associated with rs174537 genotype. This was particularly true for plasma and to a lesser extent for RBC (Figure 4; Supplementary Figure 1). This suggests that fasting circulating plasma fatty acids are more reflective of hepatic HUFA synthetic activity than are those of the RBC fatty acid profile. RBC are continuously produced as aged cells are removed. The mixture of circulating cells of heterogeneous ages, and the significantly lower RBC PUFA+HUFA content compared to plasma (RBC, 35.4 ± 11.9% total, mean ± SD; plasma 47.3 ± 4.2%total; two-tail *t*-test, *p* < 0.0001), may both contribute to placing the RBC fatty acid profile more distal from tissue sources that experience genetic governance of PUFA+HUFA synthesis compared to plasma. Nevertheless, the RBC Aggregate Desaturase Activity (ARA/LA, Figure 5C) was observed to be robustly associated with rs174537 genotype, suggesting that the DGLA and ARA content of RBC membranes may remain relatively stable over the cell lifetime. This feature may add to the value of the use of RBC fatty acid levels as indicators of longer-term *n*−6 HUFA dietary exposure.

As previously seen in cohorts of European and African ancestries, we observed that the GG genotype was associated with the highest plasma ARA and EPA (both FADS1 products) levels (Figure 4A) and the highest plasma Aggregate Desaturase Activity (ARA/LA and EPA/ALA, Figures 5A,B) in the Hispanic cohort. These biochemical phenotypes are indicative of the most efficient HUFA synthetic efficiency. However, more than 50% of the Hispanic cohort carries the TT genotype, which is associated with the biochemical phenotype of the least efficient HPUFA synthetic activity. This low efficiency HUFA synthetic phenotype, in the presence of excess dietary *n*−6 PUFAs (Figure 3; Table 2), typical of the Modern Western Diet (40), has the potential to create a *n*−3 HUFA deficiency and thus a gene-diet misalignment. Indeed, the assessment of the dietary fat exposure of this cohort suggests that their diet is reflective of the Modern Western Diet, with 7.29% of energy (17.9 ± 0.1 g/day, mean ± DEM) from LA and an LA/ALA ratio of 9.3 (Table 2). This dietary exposure and the low abundance of circulating *n*−3 PUFA+HUFA species likely contributed to our inability to unequivocally demonstrate a genetic governance of plasma estimates of *n*−3 pathway activities FADS2 (SDA/ALA), ELVOL5 (ETA/SDA) and FADS1 (EPA/ETA). Clearly, a supplementation study in a Hispanic cohort aimed at dietary ALA-enrichment and lowering of LA intake would be beneficial for better understanding the complexities of PUFA+HUFA metabolism in a low-efficiency HUFA metabolizing cohort. For example, a clinical trial prospectively comparing the capacity of a Hispanic cohort, stratified by rs174537 genotype, to metabolize an ALA enrich diet by reducing levels of LA and increasing ALA to a ratio closer to 2:1 (compared to the present 8:1) would provide great insight into the metabolic capacity of the low-efficiency metabolizing genotype. The expected result from such a trial would be a reduction in ARA levels and ARA-derived signaling oxylipins. In contrast, we postulate such a diet would elevate EPA and DHA levels and their oxylipin metabolites thereby balancing *n*−6 to *n*−3 HUFA and oxylipin levels. As with any study, there were limitations that warrant mentioning. This was a sub study of a larger trial and the parent study was not powered specifically to assess these biomarkers. The dietary questionnaire required participants to self-report the foods they consumed and therefore could have resulted in biased reporting of their dietary intake.

Beyond the biochemical phenotypes rooted in HUFA synthesis, *FADS* variants have been associated with clinical and disease phenotypes. A recent large study of healthy college-age Mexican students (37) reported that TG and VLDL levels were significantly higher for the TT genotype (48% frequency distribution) of rs174546, which is in LD with rs174537. A similar association between TG and rs174537 TT was observed in a large Hispanic cohort, with varying degrees of Amerind ancestry (17). The absence of this association (*p* = 0.083, GG + GT vs. TT) in the current study may arise from differences in health status (pre-diabetic) and the age range (18–66 years) of participants as well as a small cohort size. The former factors may also have contributed to an overall increase in blood lipid values with age.

The relationship among of PUFA+HUFA metabolism, *FADS* gene variants and anthropomorphic indices is an interesting one. Despite the relatively small cohort size of the current study, we observed a significant, negative association between the rs174537 T allele and height (Figure 6A) and a positive association with height and the *n*−6 Aggregate Desaturase Activity (ARA/LA; Figure 6B) in the female subset of the cohort. Indeed, the *FADS* locus appears to be an important site under selective and evolutionary pressures for adaptations to the presence of diet-derived nutrients that are critical for normal development and immune system integrity (11, 12, 17, 41, 42).

Overall, our findings strongly suggest a gene-diet misalignment for Hispanic/Latino populations consuming a Modern Western Diet. As a result, levels and ratios of *n*−6 and *n*−3 PUFA+HUFAs become out of balance leading to deficiencies in *n*−3 LC-PUFA+HUFAs and their metabolites, which have anti-inflammatory, cardio-protective, and triglycerides lowering properties (43–45). Even though the abundance of dietary LA exposure in this cohort (Table 2) is comparable to that assessed in other US-dwelling Hispanic/Latino cohorts (30, 46 ^1^), it is significantly lower than that of non-Hispanic Blacks and Whites (46). Nevertheless, the burden of metabolic diseases remains higher in Hispanic/Latinos populations (14, 15) and thus provides an underappreciated gene*diet feature for health disparities.

Thus, the Hispanic/Latino population, with its high frequency of the TT genotype at rs174537, is biochemically less able to synthesize the necessary HUFAs and this phenotype is exacerbated by the high LA content of the diet. The resultant *n*−3 HUFA deficiency, imposed by the gene*diet interaction, might readily be rendered less impactful if health care professionals provided nutritional guidance and education encouraging the consumption of *n*−3 HUFA-containing foods or dietary supplements. Such personalized nutrition approaches are thus potentially and pragmatically important methods to reduce health disparities.

## Data availability statement

The original contributions presented in the study are included in the article/Supplementary material, further inquiries can be directed to the corresponding author.

## Ethics statement

The studies involving human participants were reviewed and approved by Wake Forest Baptist Medical Center Institutional Review Board. The patients/participants provided their written informed consent to participate in this study.

## Author contributions

BK and SS performed the fatty acid analyses. SS isolated DNA and performed rs174537 genotyping and wrote the manuscript. JL entered and analyzed the dietary history data. SS and JL performed the data analyses. CY was the study coordinator for the intervention (parent) study. FC and MS designed the current study. SS, MS, FC, and MV edited the manuscript. MV obtained funding for the intervention (parent) study. All authors contributed to the article and approved the submitted version.

## Funding

This work was supported by grants from the National Institutes of Health, P01 MD006917 (MV) and R01 AT008621 (FC).

## Conflict of interest

FC is a co-founder of a start-up company TyrianOmega, which focuses on the production of omega-3 PUFAs by cyanobacteria, largely for animal feeds and aquaculture. He is also the co-founder of Resonance Pharma, which focuses on determining clinical level of and inhibitors that block secreted phospholipase A2 isoforms.

The remaining authors declare that the research was conducted in the absence of any commercial or financial relationships that could be construed as a potential conflict of interest.

## Publisher’s note

All claims expressed in this article are solely those of the authors and do not necessarily represent those of their affiliated organizations, or those of the publisher, the editors and the reviewers. Any product that may be evaluated in this article, or claim that may be made by its manufacturer, is not guaranteed or endorsed by the publisher.

## Footnotes

Reference 46 has been submitted and is undergoing revisions prior to acceptance for publication.

## Abbreviations

ADR: Adrenic acid
AI: Amerind
ALA: Alpha-linolenic acid
ARA: Arachidonic acid
DGLA: Dihsomo-gamma linolenic acid
DHA: Docosahexaenoic acid
DHQ1: Dietary History Questionnaire-1
DPA: Docosapentaenoic acid
ELOVL: Elongase
EPA: Eicospentaenoic acid
ETA: Eicosatetroenoic acid
FADS: Fatty acid desaturase
FAME: Fatty acid methyl ester
GLA: Gamma linolenic acid
HDL: High density lipoprotein
HOMA-IR: Homeostatic Model Assessment of Insulin Resistance
HUFA: Highly unsaturated fatty acid
LA: Linoleic acid
LD: Linkage disequilibrium
LDL: Low density lipoprotein
NAFLD: Nonalcoholic fatty liver disease
PUFA: Polyunsaturated fatty acids
RBC, SNP: Single nucleotide polymorphism
TG: Triglycerides
VLDL: Very low density lipoprotein
