# Supplementary material for: Impact of FADS gene variation and dietary fatty acid exposure on biochemical and anthropomorphic phenotypes in a Hispanic/Latino cohort
Source: Front Nutr. 2023 May 5;10:1111624. doi: 10.3389/fnut.2023.1111624 (PMC10196633; doi:10.3389/fnut.2023.1111624)
Supplement: Supplementary file 1 [file Data_Sheet_1.docx]

Supplementary Material

**Impact of *FADS* Gene Variation and Dietary Fatty Acid Exposure on Biochemical and Anthropomorphic Phenotypes in a Hispanic/Latino Cohort**

Sergeant S, Keith BA, Seeds MC, Legins JA, Young C, Vitolins MZ, Chilton FH^*^

*** Correspondence:**

Floyd H. Chilton, PhD
[fchilton@arizona.edu](mailto:fchilton@arizona.edu)

**Supplemental Figure 1** *Impact of rs174537 on n-3 PUFAs in Plasma and RBC in a Hispanic/Latino cohort*

Total plasma (**A**, top row) and RBC (**B**, bottom row) fatty acids were analyzed as described in Methods and expressed as percent of total fatty acid in the sample. Individual data are shown, stratified by rs174537 genotype (○, GG; □, GT; △, TT) for each n-3 PUFA or HUFA along the PUFA pathway. The mean and 95% CI is shown for each genotype. The data were analyzed by linear regression using an additive model for genotype, adjusting for fatty acid intake and multiple testing (Bonferroni correction, p= 0.0029). The resultant P-value for impact of genotype on each fatty acid stated under each plot.

**Supplemental Table 1:** *Genotypic Impact on PUFA Pathway Activities in Plasma and RBC*

For plasma and RBC, the Aggregated Desaturase Activity for the common enzymatic steps in n-3 and n-6 PUFA pathway and the individual enzyme surrogate ratios were evaluated for rs174537 governance. Data were analyzed by One-way ANOVA. The evaluation results are presented as the Krusckal-Wallis statistic (H), degrees of freedom (df) and the resulting P-value. An analysis was not possible when a fatty acid abundance was too low to calculate a surrogate ratio (nd, not determined) or when the enzymatic step was not applicable (na). Critical P-value corrected (Bonferroni) for multiple testing would be 0.0033 (for n= 15 endpoints). Data was derived from the entire cohort (n=135; composed of 113 females and 22 males).


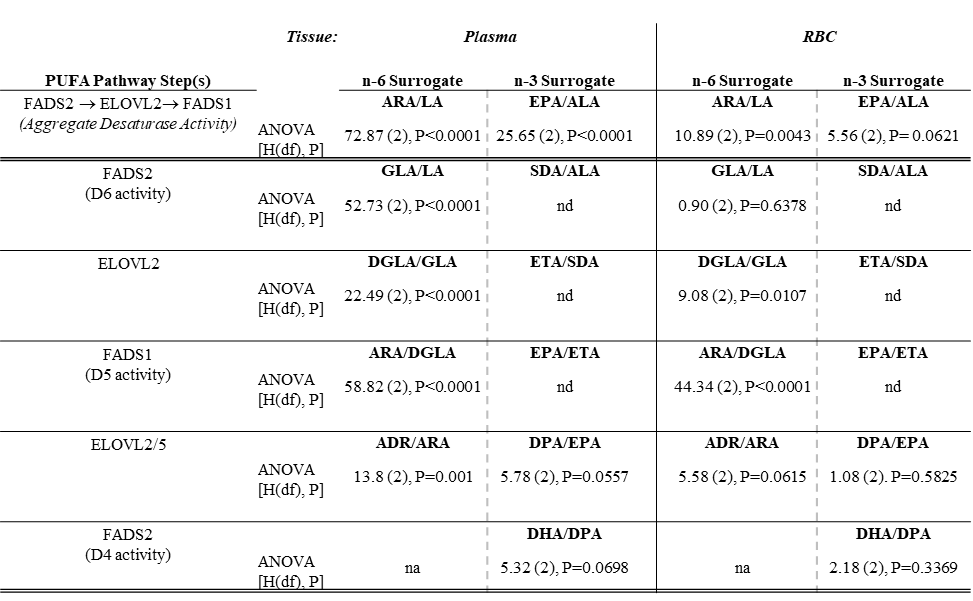


**Supplemental Figure 2** *Impact of rs174537 on the RBC Omega-3 Index in an Hispanic/Latino cohort*

The sum of RBC EPA and DHA (as % of total values) is defined as the Omega-3 Index. Individual data are shown, stratified by rs174537 genotype (○, GG; □, GT; △, TT). The mean and 95% CI is shown for each genotype. The data were analyzed by ANOVA and was found not to differ by rs174537 genotype. Data was derived from the entire cohort (n=135; composed of 113 females and 22 males).


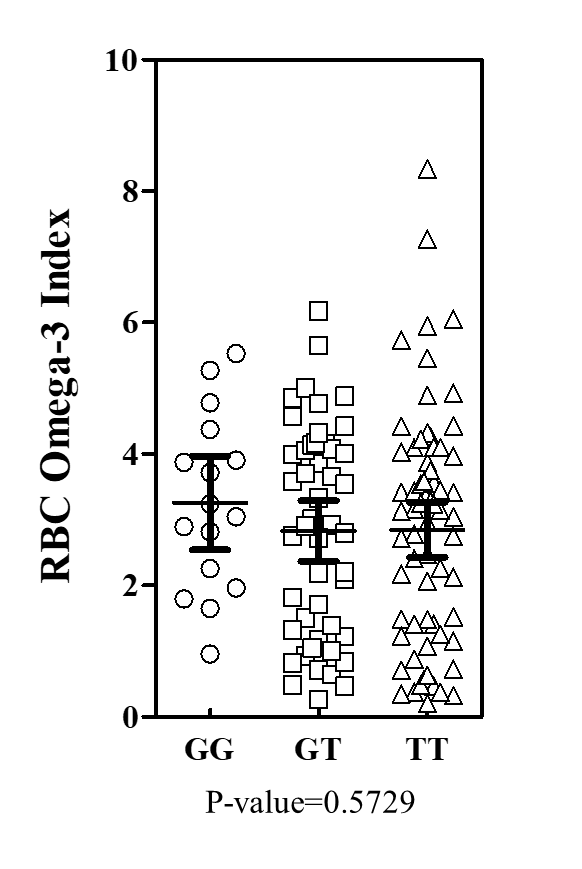


**Supplemental Table 2** *Impact of rs174537 on Clinical Phenotypes*

Age, anthropomorphic, glucose regulatory and blood lipid endpoints were analyzed by One-way ANOVA across the three rs174537 genotypes. Data shown are the mean (sd), the 95% CI and the P value. The Krusckal-Wallis statistic (H) for height had a value of 11.25.


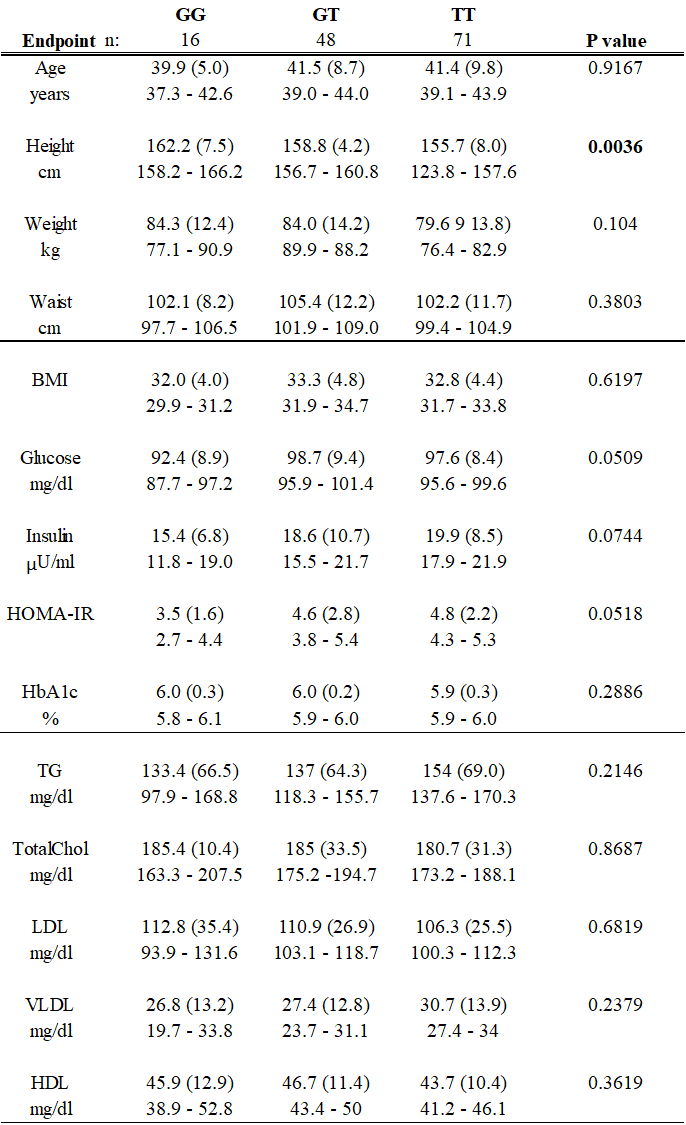


**B**
